# The Council of Emergency Medicine Residency Directors’ (CORD) Academy for Scholarship in Education in Emergency Medicine: A Five-Year Update

**DOI:** 10.5811/westjem.2016.10.31232

**Published:** 2016-11-15

**Authors:** Joseph LaMantia, Lalena M. Yarris, Michele L. Dorfsman, Nicole M. Deiorio, Stephen Wolf

**Affiliations:** * North Shore University Hospital, Department of Emergency Medicine, Manhasset, New York; † Oregon Health & Science University, Department of Emergency Medicine, Portland, Oregon; ‡ University of Pittsburgh School of Medicine, Department of Emergency Medicine, Pittsburgh, Pennsylvania; § University of Virginia School of Medicine, Department of Emergency Medicine, Charlottesville, Virginia

## BACKGROUND

In 1990, Ernest Boyer called on academic medicine to affirm its central role in education by expanding the scope of scholarship to include the domain of teaching.^1^ A decade later, Charles Glassick built on Boyer’s work by putting forth a description of six criteria for the “scholarship of teaching,” including clarity of goals, adequacy of preparation, appropriateness of methods, significance of results, effectiveness of presentation, and reflectiveness of critique.^2^ Largely in response to their work, institutionally-based academic communities, known as “academies of medical educators” came to exist in academic medicine as a means of supporting educators and promoting this newly described domain of scholarship.^3^,^4^ Over the past 15 years, these academies have grown in number with varying structures and focus, yet uniformly maintaining emphasis on the scholarship of teaching as defined by Boyer and Glassick.^1^,^2^,^3^,^4^ Currently, over 60 academies of medical educators exist in the United States, based largely at schools of medicine with some having primary affiliation with national organizations.^4^ In 2010, the Council of Emergency Medicine Residency Directors (CORD) established one of the first such specialty-based academies, the Academy for Scholarship in Education in Emergency Medicine, (EM). Details about the original inception of this academy have been described previously.^5^ This educational advance provides an update on the structure and activities of the CORD Academy for Scholarship in Education, reflecting on its first five years of service.

## OBJECTIVE

We aim to describe activities of the CORD Academy for Scholarship in Education in Emergency Medicine over its first five years of existence. In doing so, we will highlight its revised organizational structure, the evolution of the application processes, and report on accomplishments and outcomes of the Academy’s three pillars – membership and recognition; faculty development and structured programs; and education research and scholarship.

## ACADEMY DESIGN AND STRUCTURE

The CORD Academy for Scholarship in Education in Emergency Medicine was founded in 2010 as the first academy of medical educators developed within a medical education specialty society in the U.S.^5^ Since then, this model has been applied to other specialties.^6^,^7^,^8^ The mission of the CORD Academy is to promote and support excellence in education through the process of *scholarship*, as defined and described by Boyer and Glassick above.^1^,^2^,^5^ With this emphasis, the Academy strives to enhance, support, and complement the mission of CORD to advance education in EM.

At its beginning, the Academy was a service organization with loose organizational structure. Members were solely “distinguished educators” (DE) chosen annually through a criterion-based, peer-reviewed selection process in one or more of four categories based on the scholarship of teaching: teaching and evaluation, enduring educational materials, educational leadership, and educational research.^2^ By way of service, DEs would contribute their expertise and skill through a variety of programs and initiatives related to faculty development, mentoring, consultation, liaison work, and advocacy, striving to advance education through scholarship among both the Academy and the entire CORD membership. In 2014, as the Academy grew in membership a systematic organizational review process was undertaken, yielding a new organizational structure (Figure). This process reaffirmed three *pillars* of the Academy’s mission (i.e., membership and recognition; faculty development and structured programs; and educational research and scholarship) and laid the groundwork for tiered membership (i.e., DE, academy scholar, and academy member). All members are encouraged to align with a pillar, in part to meet their service obligation and assure progress on yearly pillar objectives.

Furthermore, under this new structure, termed leadership positions were established. Currently, an academy director, immediate past-director, and three pillar leaders oversee the business of the Academy, determine and execute annual strategic objectives for the Academy, and act as liaisons to the CORD Board of Directors regarding the activities of the Academy. An Academy Advisory Committee provides long-term direction and vision, as well as advice and counsel to the Academy director on the administration and annual strategic objectives. This Advisory Committee is composed of the CORD Board of Directors president-elect, two at-large CORD Members, the chair of CORD’s Academic Assembly Advisory Committee, and Academy leadership. The Academy members, Academy leadership, and the Advisory Committee each meet regularly to assure adherence to the Academy’s goal and mission^5^ and progression of annual strategic objectives.

## PILLAR ROLES AND EFFECTIVENESS

The CORD Academy’s effectiveness can be measured in terms of accomplishments and success of each of its three pillars. Less quantifiable is its success as determined by its value to its members and the general CORD membership as a community of practice and network for educators seeking to promote the scholarship of teaching. This latter success will be borne out over time as the academy grows and matures.

### Membership & Recognition

The Academy’s Membership & Recognition pillar is tasked with reviewing new applications to the Academy and tracking recurrent eligibility for existing members. Membership is criterion-based, and applicants must demonstrate they have met standards as embodied by a set of example applications. Example applications exist for various types of potential applicants, such as those with undergraduate medical education (UME) or GME-focused careers. The application preparation process is rigorous, but intended to objectively help the applicant demonstrate excellence in their selected area of focus (i.e., Teaching and Evaluation; Enduring Educational Materials; Educational Leadership; and Education Research). A complete application consists of eight parts: a letter of submission, a match to standard setting examples, structured summary, personal statement, structured abstract, table of appendices, supporting documents, and a curriculum vitae.^9^ Potential applicants may ask for an Academy mentor to assist with the application process and periodic consultation sessions are offered at the CORD Academic Assembly. Applications are called for annually, and the pillar members use a systematic, criterion-based group review and scoring process to determine candidacy.

Until recently, and similar to academies of medical educators across the country, membership in the Academy was conferred through a DE award process. However, in 2014, in order to reach a broader potential membership and create a more vibrant group of faculty, membership categories were expanded to now include three criterion-based tiers - distinguished educator, Academy scholar and Academy member. Currently, DEs are selected as members who have demonstrated the highest level of commitment to and excellence in medical education as evidenced by significant quality, quantity, and breadth within their chosen area of focus. Selection of scholars is based upon their attaining recognition as remarkable educators who have demonstrated a significant level of commitment and excellence within a chosen area of focus. Finally, Academy members are selected by their stated commitment to education and scholarship and willingness to actively participate in the Academy as a service organization.

A Legacy Mentor category was also introduced in order to include senior faculty, advanced in their academic career, who might not otherwise submit a comprehensive application. Those wishing to apply for this designation submit an abbreviated, yet still criterion-based, application. Once a part of the Academy, members are expected to affiliate with a pillar and contribute by participating in service to their pillar. Currently, members must renew their application every 3–5 years, depending on their level of membership. The effectiveness of the Membership & Recognition pillar is marked by the continued submission of high quality applications resulting in 30 current members.

### Faculty Development and Structured Programs

Faculty development is the heart of the Academy’s mission, with the hopes of creating scholarship from each endeavor in collaboration with the Education Research and Scholarship pillar. The most well known program under this pillar is the CORD Coaching Program.^9^ This structured, three-step peer-to-peer mentoring program is designed to assist national speakers at all levels to improve their introspection, confidence, and teaching innovation. Following structured self-reflection and observation sessions, assigned academy DEs provide feedback and recommendations aimed at helping speakers to overcome identified challenges, improve their skills and develop their careers. To date, the CORD Coaching Program has conducted over 30 coaching sessions for CORD members, uniformly generating positive feedback from participants. Future efforts will need to focus on formally assessing the impact of the program on teaching skills of participants.

An additional Academy initiative currently underway is a collaboration between the Faculty Development Structured Programs and the Education Research and Scholarship pillars. Both groups are working to develop a critical appraisal and annotated journal club series. This series aims to identify key medical education topics that are relevant to EM educators, perform a critical appraisal of the literature to identify leading papers on that topic, and summarize current understanding. Examples of potential topics for this program include fundamental teaching skills, feedback, student remediation, and producing educational scholarship.

The Faculty Development and Structured Programs pillar also strives to enrich the educational offerings of the CORD Academic Assembly and other national EM meetings. Sessions on educational portfolio development, coaching, mentoring, promotion and tenure, educational consults, promotion of scholarship at home institutions, and various teaching skills are presented annually by Academy members, scholars and DEs. To date, Academy members have sponsored over 20 faculty development and 10 one-on-one education portfolio development sessions for CORD members and other EM educators at national meetings. While these sessions and the above coaching activities have been well received, future efforts of the Academy will focus on explicitly measuring the effectiveness of these sessions.

Lastly, this pillar is investigating ways to share educational materials, such as lectures and small group sessions, with a plan to allow for credit to be given to authors. This is similar to the Med-Ed Portal model as it applies to national didactics made portable.

### Education Research and Scholarship

The Education Research and Scholarship pillar seeks to support educators in their scholarship endeavors, promote EM education research, and move the science of medical education forward. The pillar aims to serve as a virtual “community of practice” for EM education researchers, and has envisioned four main components:

- Grants & Scholarships – This pillar has worked with the CORD Board of Directors and the Emergency Medicine Foundation to develop the joint EMF/CORD Emergency Medicine Education Research Grant, providing up to $25,000 funding to qualifying proposals to study medical education topics with direct relevance to the specialty of EM.^10^
- Research Faculty Development Programs – In 2015–2016, the position of Director for Professional Development in Education Research was created. This leader will collaborate with the Faculty Development and Structured Programs pillar to specifically address CORD members’ faculty development needs in education research and scholarship. This pillar also supports the Medical Education Research Certificate (MERC) at CORD Scholars Program (MCSP), as well as Academy sessions at national meetings that focus on education research skills. MCSP has experienced significant success in developing over 150 clinical-educators in education research methodology and skills. 11
- Education Research Consortium – The Academy aims to build upon the prior work done to establish guidelines and a structure for a collaborative education research consortium, previously called Emergency Medicine Education Research Group (EMERGe).^12^ The pillar envisions that the consortium would serve as both a central resource for multicenter study administration and a structure to encourage collaboration.
- CORD Center for Program Evaluation and Learner Assessment – A future direction includes a proposed entity that would serve as a resource for design, methods, and statistical expertise to assist with program evaluation and learner assessment efforts that serve the CORD community. The center, for example, could contribute to national needs assessments, CORD educational program evaluation, and as a consult for EMERGe studies that aim to develop and test assessment tools. Existing CORD task forces and committees with aligned missions could fall under the Center for Program Evaluation and Learner Assessment, such as the Joint Milestone Task Force and the Systematic Evaluation Methods committee.

The Education Scholarship pillar is in the process of completing a national workforce study of EM educators that intends to describe the current landscape of educational program administration and staffing, as well as a needs assessment to identify strategies that would most help EM educators reach their education research aspirations.^13^

## CHALLENGES

The CORD Academy for Scholarship in Education in Emergency Medicine worked to overcome multiple structural and functional challenges. Structurally, as an organization based within a medical education specialty society, the CORD Academy has had an opportunity to promote educational excellence on a national level, using the resources of its parent organization. However, its organizational affiliation may serve as a barrier to broad integration across all EM educators, some of whom may identify with other national EM organizations. In the planning and inception of the Academy, efforts were made to secure the strong support and commitment of CORD as one of the lead organizations in EM education. As envisioned, the CORD leadership has been able to advocate for the Academy, promoting its unique benefits to the CORD membership and other EM educators.

Another challenge the Academy has faced is assuring that the development of our educational innovations, services and offerings do not outpace the available resources of our members’ service commitment or our available financial and administrative support. Academy members, scholars and DEs are accomplished and committed individuals. The Academy continuously works to balance member service obligation and resources utilization to provide added worth to its membership and the CORD organization as a whole. It is our hope that this usefulness will continue to support the Academy’s credibility and purpose both within CORD and to EM educators.

Next, the ideal balance of inclusivity of membership criteria and selection has proven to be a challenge to the Academy’s growth. The Academy was rigorously selective in its early cycles of membership; however, feedback led to significant efforts to broaden membership and foster inclusivity so as to provide greater value to the whole of the CORD membership.

Finally, it is important for the success and viability of the Academy to have continuity of purpose and process, and sound organizational memory. It is our hope that in future years, as the Academy continues to grow, the prescribed roles of the Academy Advisory Committee, Academy leadership positions and overall organizational structure of the Academy will provide this historical perspective and memory.

## SUMMARY

After five years, the CORD Academy for Scholarship in Education in Emergency Medicine is gaining traction as a national academy for EM health professions educators. The Academy has worked to overcome structural and functional challenges in order to operate as a service organization and community of practice within the CORD. Future efforts will continue to focus on providing value to educators by recognizing excellence, promoting career development, and fostering the scholarship of teaching in emergency medicine.

## Figures and Tables

**Figure:**
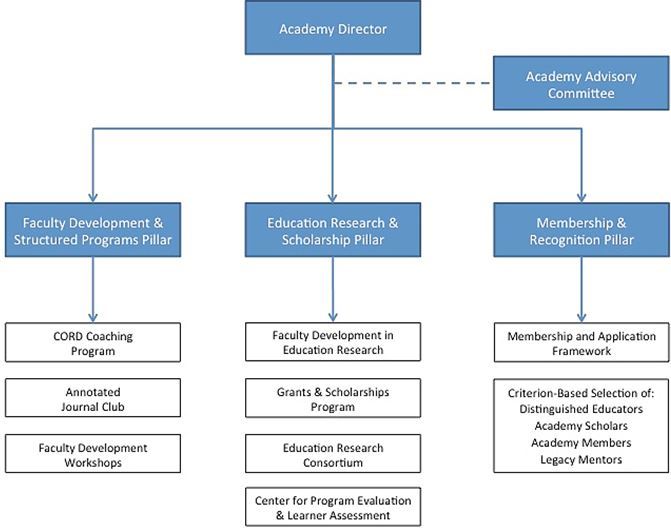
Council of Emergency Medicine Residency Directors’ (CORD) Academy for Scholarship organizational structure.
